# Oncologic Outcome after Pulmonary Metastasectomy as Part of Multidisciplinary Treatment in a Tertiary Oncological Center

**DOI:** 10.3390/diagnostics13010165

**Published:** 2023-01-03

**Authors:** Natalia Motas, Mihnea Dan Davidescu, Bogdan Cosmin Tanase, Ovidiu Rus, Alin Ionut Burlacu, Vlad Alexe, Veronica Manolache, Madalina Cristiana Mizea, Nicolae Gheorghiu, Oana Gabriela Trifanescu, Laurentia Nicoleta Gales, Teodor Horvat, Rodica Maricela Anghel

**Affiliations:** 1 Clinic of Thoracic Surgery, “Carol Davila” University of Medicine and Pharmacy, 050474 Bucharest, Romania; 2 Department of Thoracic Surgery, “Prof. Dr. Al. Trestioreanu” Institute of Oncology Bucharest, 022328 Bucharest, Romania; 3 Department of Thoracic Surgery, Memorial Oncology Hospital, Șoseaua Gheorghe Ionescu Sisești 8a, 013812 Bucharest, Romania; 4 Clinic of Oncology, “Carol Davila” University of Medicine and Pharmacy, 050474 Bucharest, Romania; 5 Department of Radiotherapy II, “Prof. Dr. Al. Trestioreanu” Institute of Oncology Bucharest, 022328 Bucharest, Romania; 6 Department of Medical Oncology II, “Prof. Dr. Al. Trestioreanu” Institute of Oncology Bucharest, 022328 Bucharest, Romania

**Keywords:** pulmonary metastases, lung metastasectomy, MITS minimally invasive thoracic surgery, uniportal VATS video-assisted thoracic surgery, metastatic cancer, LASER metastasectomy, metastatic colorectal cancer, metastatic breast cancer, metastatic lung cancer, metastatic sarcoma

## Abstract

(1) Background: Pulmonary metastases are encountered in approximately one-third of patients with malignancies, especially from colorectal, lung, breast, and renal cancers, and sarcomas. Pulmonary metastasectomy is the ablative approach of choice, when possible, as part of the multidisciplinary effort to integrate and personalize the oncological treatment. (2) Methods: The study includes 58 consecutive cases of pulmonary metastasectomies, retrospectively analyzed, performed in 12 consecutive months, in which the pathology reports confirmed lung metastases. (3) Results: Most frequent pathological types of metastases were: 14 of colorectal cancer, 10 breast, 8 lung, and 8 sarcomas. At the time of primary cancer diagnosis, 14 patients (24.14%) were in the metastatic stage. The surgical approach was minimally invasive through uniportal VATS (Video-Assisted Thoracic Surgery) in 3/4 of cases (43 patients, 74%). Almost 20% of resections were typical (lobectomy, segmentectomy). Lymphadenectomy was associated in almost 1/2 of patients and lymph node metastases were found in 11.11% of cases. The mortality rate (intraoperative and 90 days postoperative) is zero. The OS after pulmonary metastasectomy is 87% at 18 months, and the estimated OS for cancer is 90% at 5 years. The worst outcome presents the patients with sarcomas and the best outcome—colorectal and lung cancer. The patients with 1 or 2 resected metastases presented 96% survival at 24 months. (4) Conclusions: After pulmonary metastasectomy, survival is favored by the small number of metastases resected (1 or 2), and by the dimension of metastases under 20.5 mm. The non-anatomic (wedge) type of lung resection may present a lower risk of death compared to lobectomy. No statistical significance on survival has the presence of lymphadenectomy, the laterality right/left lung, the upper/lower lobes. In the future, longer follow-up and prospective randomized trials are needed for drawing definitive conclusions.

## 1. Introduction

Pulmonary parenchymal tissue represents a common site for metastatic seeding, the lung being a filter for the whole circulation. Approximately one-third of patients with a malignant disease will develop pulmonary metastases. The tumors that have an affinity for lung parenchyma are colorectal cancers, sarcomas, lung cancers, breast, renal cell, and head and neck carcinoma [1,2,3].

Management of pulmonary metastases is a multidisciplinary effort to integrate and personalize surgery, systemic treatment, and alternative therapies. Immunotherapy and targeted therapies impact the strategies. Factors as primary tumor status, the natural history of the disease, pathology, genotype, and availability of effective treatment are considered. Immunotherapy and targeted therapies are applicable in melanoma, or renal cancers, and surgical resection is reserved as consolidation or as salvation therapy in non-responsive lesions.

Surgical resection is considered a valid part of the multimodal treatment of pulmonary metastases representing today a significant portion of the activity of any thoracic surgery department. According to the database annual report of the European Society of Thoracic Surgeons, pulmonary metastases accounted for 10.2% of all resected lung pathologies in 2021 [4].

Although more than 1000 articles are being published on pulmonary metastasectomy, there are practically no randomized controlled trials. Case series are being reported on fit patients, with a lower number of metastases and less aggressive primary tumors. No comparative survival analysis can be performed because of missing data on non-surgical treated pulmonary metastases. Historical controls are cited instead on the survival of patients with pulmonary metastases not treated by surgery.

In the era in which more and more treatments for cancer appear, the role of surgery in the treatment of lung metastases must be re-evaluated. Another issue in this context is the increasing need for re-biopsy in non-resectable metastases, for adequate adjustment of systemic therapy.

The goal of these interventions is to cure or prolong life expectancy, but we have to keep in mind that pulmonary metastasectomy is local therapy for a, theoretically, nonlocalized disease.

According to the “Expert Consensus Document on Pulmonary Metastasectomy” (2019), the selection of patients for pulmonary metastasectomy includes the control of primary site and the active management of other metastases, if any [5]. In those patients, the imaging modalities and the risk assessment including performance status function “does not differ from that of a patient evaluation for medical operability of primary lung cancer” [5]. The same consensus states that there is no literature guidance regarding timing for pulmonary metastasectomy relative to completion of systemic therapy [5].

In the absence of new randomized trials, the expert consensus and the clinical guides recommend pulmonary metastasectomy for selected patients within a multidisciplinary team management [5].

The aim of this study is to evaluate the surgical approach to pulmonary metastases in terms of overall survival, number of resected metastases, type of primary cancer, and type of resection.

## 2. Materials and Methods

Between March 2021 and February 2022 (12 months), 67 consecutive surgical approaches on suspected lung metastases were performed on 64 patients. In 3 patients, a bilateral sequential surgical approach was performed.

Of the total of patients (more than 300) addressed for lung metastases, the majority were recommended for non-surgical treatment, and only for 64 patients, surgical approach was indicated.

**Figure 1:**
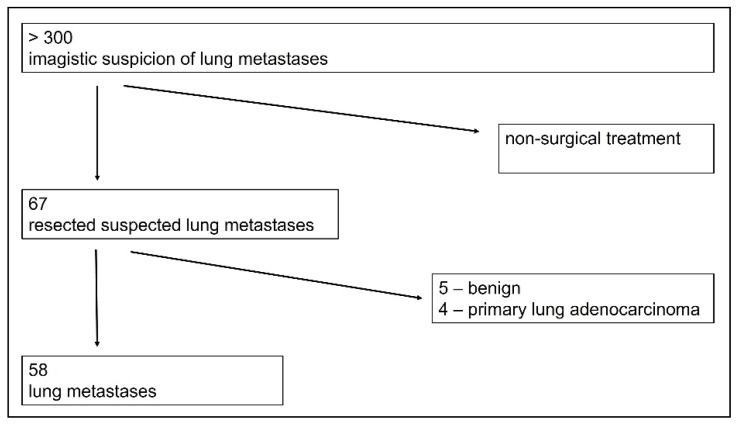
Inclusion criteria for patients in the study—selection from the presenting patients with suspicioned lung metastases.

Before pulmonary metastasectomy, local + systemic therapy for the primary site had been performed according to guidelines and protocols and in accordance with the initial staging, except the cases in which the primary tumor was diagnosed in the metastatic stage and a pathological conformation of the lung nodules (inaccessible by non-surgical biopsies) was necessary for initiating the systemic oncological treatment. The indication for surgical intervention in those cases was decided by the multidisciplinary board. All patients submitted their written consent for treatment, according to the local policy. Pathology reports showed lung metastasis in 58 patients and other histology in the rest of the 9 patients (Figure 1).

Fifty-eight patients were included in our study and retrospectively analyzed, based on the pathological result of resected pulmonary metastasis, completeness of clinical data, and presence of follow-up. Exclusion criteria were the pathological report showing either a benign result of the resected pulmonary lesion or a primary pulmonary malignant histology (another cancer besides the known one), non-surgical treatment of the pulmonary metastases.

The statistical analysis was realized with SPSS, version 23.0 for Windows, and included all eligible patients. The oncologic outcome was reported using the Kaplan–Meier method to determine progression-free survival after metastasectomy define as the time from metastasectomy to the disease progression on imaging (according to RECIST 1.1) or death from any cause and cancer median overall survival defined as the time from diagnosis to death of any cause and metastasectomy overall survival define as time form metastasectomy to death from any cause. The univariate analysis using the log-rank test was used for studying the influence of different factors regarding the oncologic outcome, and time to disease progression, and multivariate analysis was used according to the stepwise Cox proportional hazards model to identify independent prognostic factors and estimate their effect on the time to disease progression and overall survival. The confidence interval (CI) considered for the calculated quantitative variables was 95%, and the *p*-value considered statistically significant was <0.05. Receiver Operating Characteristics (ROC) curves were used to measure the model’s efficacy, determine a prognostic cut-off value, and estimate the sensibility and specificity of the method. An AUC (Area Under the Curve) closer to 1 is considered an efficient model and AUC values > 0.6 validate the model.

The study was approved by the Ethical Committee of the Institute of Oncology (24912/2022). No specific Informed Consent Form (ICF) was used because all patients signed the Institutional ICF giving consent to full use of their medical records for research purposes. The study was conducted in harmonization with the World Medical Association (WMA) Helsinki Declaration of 1975, as revised in 2008.

## 3. Results

### 3.1. Patient Characteristics

Of the 58 patients, 40 were female (68.9%) and 18 were male (31.1%), with an average age of 59.7 ± 11.81 years, range 28–80 years. 

Types of primary cancer are presented in Table 1. Most frequent are colon (including rect) cancers followed by breast malignancies. The group with sarcomas contains two leiomyosarcomas, two osteosarcomas, one uterine carcinosarcoma, one uterine sarcoma, and one Ewing sarcoma. There was one patient with a history of rectal GIST (Gastro-Intestinal Stromal Tumor), with bilateral sequential pulmonary metastasis resected.

At the time of diagnosis of primary cancer, 14 patients out of 58 (24.14%) were in the metastatic stage.

Before lung surgery, the usual clinical exam and paraclinical investigations were performed; PET-CT was performed according to the multidisciplinary board recommendations, considering each patient’s particularities, e.g., stage of cancer at the diagnostic moment, treatment received for primary cancer, histologic type of cancer, comorbidities. The “Expert Consensus Document on Pulmonary Metastasectomy” (2019) recommends as imagistic preoperatory work-up the CT-scan for “number, location and technical resectability” of pulmonary metastases and the PET-scan “for the extrathoracic disease, if the primary tumor is avid” [5].

The surgical approach was minimally invasive in 3/4 of cases (43 patients, 74%), and in 15 patients (26%) a classic open thoracic surgery access was performed (by thoracotomy) imposed by each case particularities. The minimally invasive thoracic surgery MITS approach was uniportal VATS (video-assisted thoracic surgery) in all 43 patients.

As the type of resection (Table 2), typical lung resection (lobectomy, bilobectomy, or segmentectomy) was performed in 11 cases. No pneumonectomy was performed.

Most of the patients (47 from 58, 81%) needed wedge lung resections (atypical, non-anatomical lung resections). Associated resections imposed by tumoral extension and necessary for the completeness of excision are presented in Table 2.

In 36 cases, the resected metastasis was unique—62%; in 12 cases (20.68%) two metastases were resected. In the rest of 10 cases (17.32%) the number of metastases resected was more than 2. The maximum number was 15 metastases resected (in one patient with metastasis from colonic cancer, alive and free of disease at 15 months after pulmonary metastasectomies).

The total number of surgically approached metastasis was 116, with an average number of 2 metastases. The average dimension of the resected metastases in all 58 patients was 17.11 mm, the range being 3–140 mm.

Lymph node approach was performed in almost half of the patients (27 patients, 46.5%), with no lymphatic approach in the rest of the 31 patients. All anatomic lung resections (11—typical, lobectomy, or equivalent) were completed with lymph node dissection. In wedge lung resections, a lymph node approach was performed (16 cases) either when the adenopathy’s were discovered intraoperatory, or when decided by the surgeon. Pathologic reports showed lymph node metastasis in 3 patients: 1 patient with N1 hilar station (from cervix cancer) and 2 patients with N2 mediastinal cancer (from colonic and, respective, renal cancers). Lymph node metastasis was found in 3 cases from 27, representing 11.11%.

Postoperative complications appeared in four patients, in three cases being related to the surgical intervention (prolonged air leak in two cases and intrapulmonary small hematoma in one case) and in one case non-related to the lung surgery (bilateral bronchopneumonia necessitating ventilatory support); so, the postoperatory morbidity was 6.89%.

Intraoperatory, immediate and late postoperative mortality rates (30 days, 90 days) were zero (0%).

### 3.2. Overall Survival (OS) after Metastasectomy

**Figure 2:**
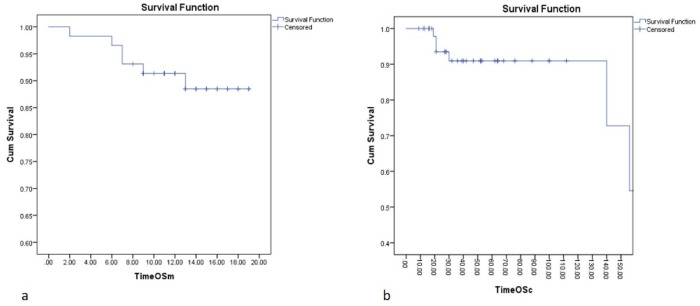
Overall survival of patients after metastasectomy (**a**) and from the diagnostic of cancer (**b**).

The median follow-up after pulmonary metastasectomy was 13 months. The median OS was not reached. The mean OS was 17 months. Estimated OS at 6 months is 96%, at 12 months is 92% and at 18 months is 87% (Figure 2a).

Overall survival for cancer results are: the median OS is 149 months. A 5 years median OS is of 90%, with median follow-up for cancer survival of 40 months (Figure 2b).

**Figure 3:**
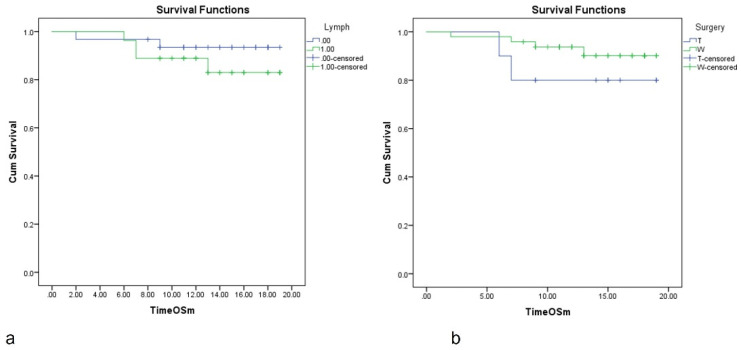
Survival analysis according to the presence of lymphadenectomy associated with the pulmonary resection (**a**) and according to the type of lung resection: T = typical resection (anatomic resection), W = wedge resection (non-anatomic, atypical resection) (**b**).

Survival analysis according to lymphadenectomy shows no statistical difference between lymphadenectomy and without lymphadenectomy—the 12 months OS was 88% for lymphadenectomy vs. 94% for patients without lymphadenectomy (Figure 3a).

Regarding the type of resection, in COX regression analysis typical resection (lobectomy) was associated with higher risk of death of HR = 1.831, 95% CI, 0.334–10.031, *p* = 0.486, comparing to wedge resection (non-anatomical lung resections); the result is lacking statistical significance. Median survival was not reached. The estimated mean survival in patients with typical (anatomical) lung resection vs. wedge (non-anatomical) was 16.5 months vs. 17.96 months (Figure 3b).

Regarding the number of metastases excised, the best survival was for 1 or 2 metastases—those patients present 96% survival at 24 months; for 3 metastases excised the survival is 65% at 36 months; worst survival is for 4 metastases: 70% at 12 months and 35% at 24 months. The worst prognosis is for sarcomas and cervical cancer. 

**Figure 4:**
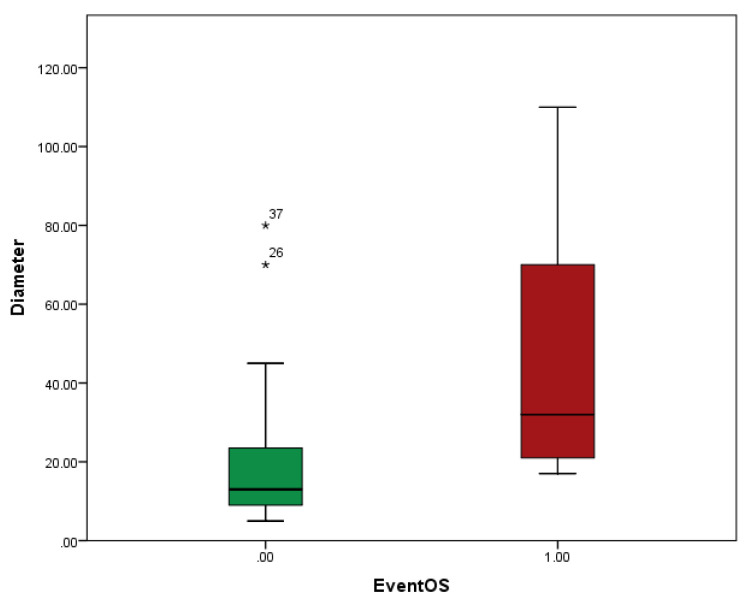
Analysis of the dimension of metastases regarding the fatal event.

There was a statistical difference between the mean diameters of the largest metastasis excised in patients in which the event happened (death) or patients still alive (47 mm vs. 18 mm, *p* = 0.001) (Figure 4), emphasizing the importance of the accurate selection of patients and early referral to thoracic surgery. There was no difference in the mean number of metastases excised in dead or alive patients (*p* = ns).

**Figure 5:**
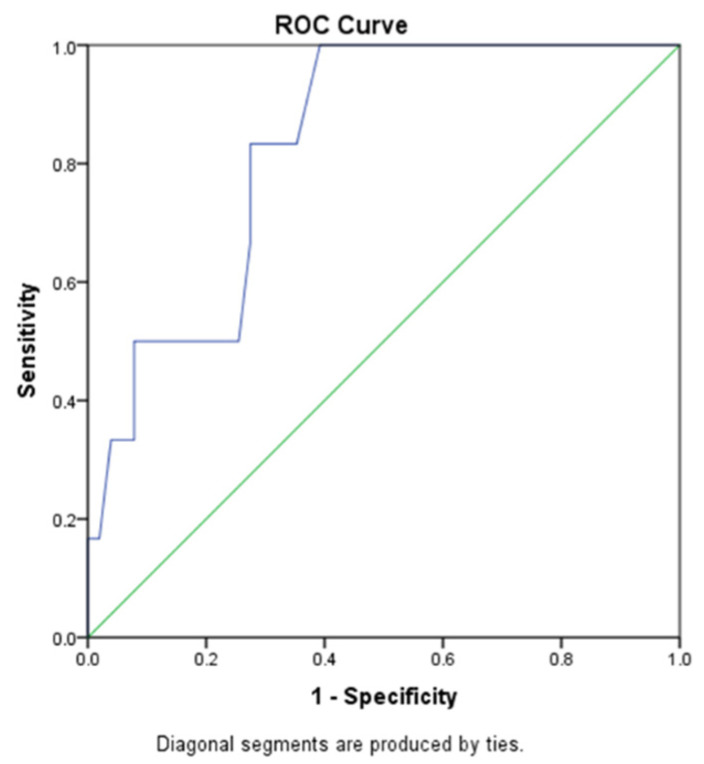
Analysis of the effect of the resected metastases dimension on oncologic outcome.

In order to evaluate the effect of tumor dimension after metastasectomy on the oncologic outcome, Receiver Operating Characteristics (ROC) curves were used. Thus, the area under the curve of tumor dimension for the estimation death was 0.830, *p* = 0.009, 95% CI 0.69–0.965) The cut-off value of dimension to predict death with 83% sensitivity and 27% specificity was 20.5 mm (Figure 5).

There was no statistically significant difference in estimated survival in patients presenting right and left metastases, or in upper and lower lobes, or comparing upper, medium, and lower lobes.

**Figure 6:**
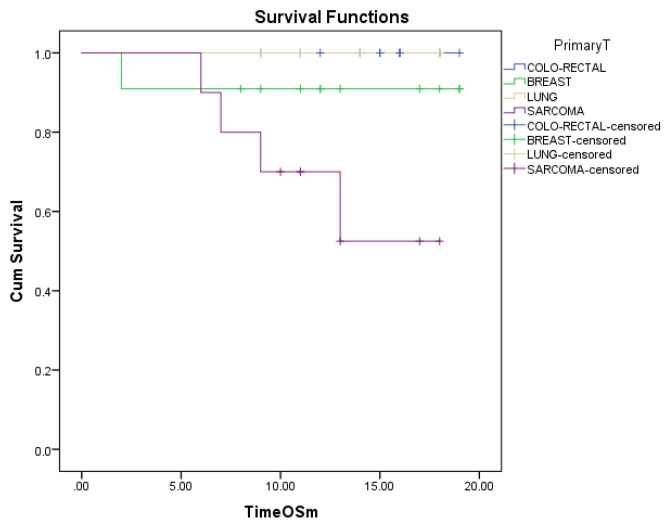
Overall survival of the first four most frequent types of cancers from the lot of study.

Regarding the overall survival of different primary tumors after metastasectomy, the worst outcome was noticed in patients with sarcoma, and no death was yet reported in patients with colorectal and lung cancer. The COX regression analysis showed a statistically significant worst outcome for sarcoma *p* = 0.04 with an increase of risk of death of 3.4 times for patients with sarcoma compared to other sites. (HR = 3.45, 95%CI 1.056–11.286) (Figure 6). Longer follow-up is needed. 

## 4. Discussion

Despite a large literature, more than 1000 articles being published on the subject of pulmonary metastasectomy, there are practically no randomized controlled trials. The reported surgical case series manifest pervasive selection bias: by choosing the more fit patients (that can withstand the operation), those with fewer metastases, and less aggressive tumors. There are no comparable data on the patients who did not have metastases removed so no comparative survival analysis can be performed. Historical controls are cited instead on the survival of patients with pulmonary metastases not treated by surgery. 

Another confounding factor is the use of systemic therapy before or after the operation, which may influence the outcomes, raising the possibility of reverse causation—the metastasectomies could be performed because of the longer survival of the patients. One of the more powerful examples is the long-term prognostic analysis of 5206 lung metastasectomies from the International Registry of Lung Metastases in 1997, which endorsed pulmonary metastasectomy as a therapeutic option in current practice [6].

A systematic review of the literature made in 2021 by Kai-Yin Lee and collab. [7] identified only six studies that compared survival outcomes of lung metastasectomies in colorectal cancers—two randomized controlled trials, based on the same database and 4 retrospective cohort studies. So far, a single randomized clinical trial has been performed: the Pulmonary Metastasectomy versus Continued Active Monitoring in Colorectal Cancer (PulMiCC) trial, which was stopped early because of poor recruitment [8]. In this trial, the 5-year survival of the operated patients was 38%—in line with the existing literature but the survival of the matched control patients was better than expected with 29%. The small number of patients enrolled in the trial (65 patients) prevents a definitive conclusion.

We are contemporary with a myriad of new treatments for cancer and the role of surgery in the treatment of lung metastases must be reassessed. Surgical resection of pulmonary metastases overlaps with the oligometastatic disease and, at least partially, with the salvage surgery concept. The desiderate of lung metastasectomy is a patient free of disease with long survival and (at least) good quality of life.

Generally, the preoperative histologic confirmation of lung metastases may be either impossible or too risky compared to the benefits, so the diagnostic is affirmed on imagistic findings and/or imagistic evolution. In such situations, surgical excision remains the only solution and solves both diagnostic and treatment problems.

In the absence of new randomized trials, the “Expert Consensus Document on Pulmonary Metastasectomy” (2019), the clinical guides, and publications from the literature recommend pulmonary metastasectomy for selected patients within a multidisciplinary team management [5,9,10,11,12,13,14,15,16,17,18,19].

The established patient selection criteria for pulmonary metastasectomy are: (I) primary tumor control (II) no other extra-thoracic metastases or they can be controlled, (III) complete metastasis resection technically possible, (IV) the patient must be able to tolerate the resection, (V) no better alternative therapy [1,20]. Another emerging indication refers to rogue metastasis or oligoprogression in which a few metastases progress while other sites appear to be well-controlled and inactive. They can be managed with local therapy ablation, allowing the maintenance of a still effective systemic therapy, also known as treatment beyond progression [21,22].

Other indications for pulmonary metastasectomy, besides those with curative intent, are: confirmation of the diagnosis in patients with previous neoplasia, who presents with pulmonary nodules on control CT scans; excision of a residual mass after chemotherapy; providing suitable metastatic tissue for histopathological analysis for targeted therapy or immunotherapy; reduction in tumor burden for secreting tumors such as parathyroid cancer, and, rarely, relief of symptoms such as hemoptysis [2,5,23].

The main goal of lung metastasectomies is to achieve a complete resection of the tumors while preserving as much pulmonary parenchyma as possible. Lung tissue should be saved to preserve the quality of life and allow reresections if needed.

The extent of pulmonary resection is dictated by the need to achieve a radical resection. Wedge resection and enucleation by LASER are still the preferred interventions because they permit preservation of lung parenchyma and re-resections in case of relapse.

Enucleation by LASER is best in preserving pulmonary parenchyma followed by wedge resection. The laser can be deployed mini-invasively by using the naked fiber but at a lower power setting which leads to a longer resection time. Smoke is also a problem in the VATS application of LASER. On the other hand, the laser, because of the higher precision and narrower resection margins, allows the excision of centrally located tumors that otherwise would require typical resections.

The current practice is highly endorsing minimally invasive procedures so wedge resection has become the dominant surgical approach for pulmonary metastases [24].

Identification of small, deep, pulmonary nodules raises certain challenges. Careful study of CT scans helps guide surgeons. Finger palpation through ports or instrumental palpation are helpful in bigger or more superficial nodules. For smaller deeper nodules different localization techniques have been described: preoperative hook-wire placement, methylene blue injections, percutaneous coils, injection of radioactive isotopes, and intraoperative identification with a portable sensor [5,23,25]. Conversion to thoracotomy should be considered if the nodule cannot be identified. Besides the well-documented advantages of minimally invasive approaches, an important advantage is the formation of fewer pleural adhesions than in open techniques which permit repeat metastasectomies in case of recurrence [1,20].

In our study, 3/4 of surgical approaches were minimally invasive—VATS (43 patients, 74%). No additional identification method (besides intraoperative instrumental palpation) was necessary (for example, the hook in the nodule placed under CT scan guidance, as it was performed in several cases outside the period of this study) and no conversion to thoracotomy for identifying small metastases.

Historically, manual palpation via thoracotomy has been considered as a standard surgical approach necessary to avoid missing the metastases when multiple are found on preoperative radiological examinations. Bimanual palpation permits the identification of small nodules that otherwise would be missing. Some studies have shown that additional metastases can be found if thoracotomy is performed after VATS resection [26]. However, modern high-resolution CT scanners can identify minute nodules in the lung, making the possibility of finding others by palpation very slim. Moreover, retrospective studies suggest that the open approach with its improved detection and resection rates does not lead to improved survival after surgery and the overall survival and recurrence survival did not differ between VATS and open approaches, irrespective of the type of the primary tumor [1,20].

**Figure 7:**
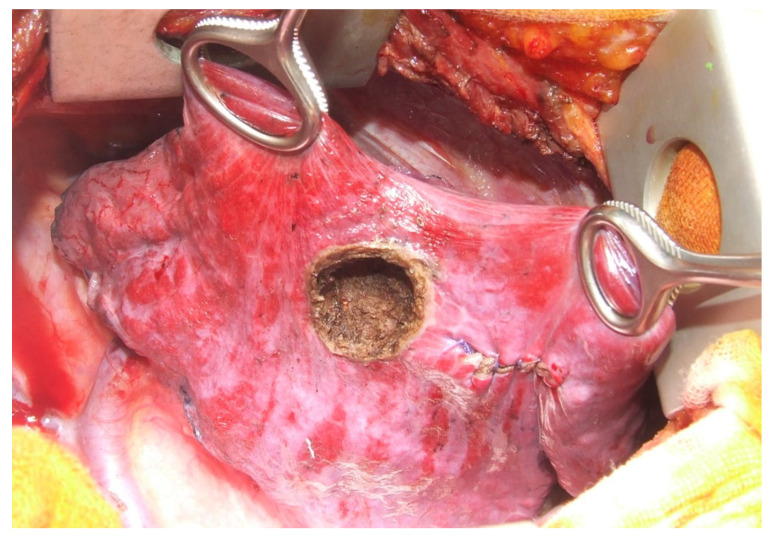
LASER pulmonary metastasectomies performed through open thoracic surgery.

In our study, LASER metastasectomy and LASER ablation of metastases were performed in 10 patients (Figure 7). For palpation and identification of the metastases, it was planned and performed open access (thoracotomy), knowing the fact that intraoperative the number of metastases to be resected is usually bigger than on the preoperatory CT scan.

**Figure 8:**
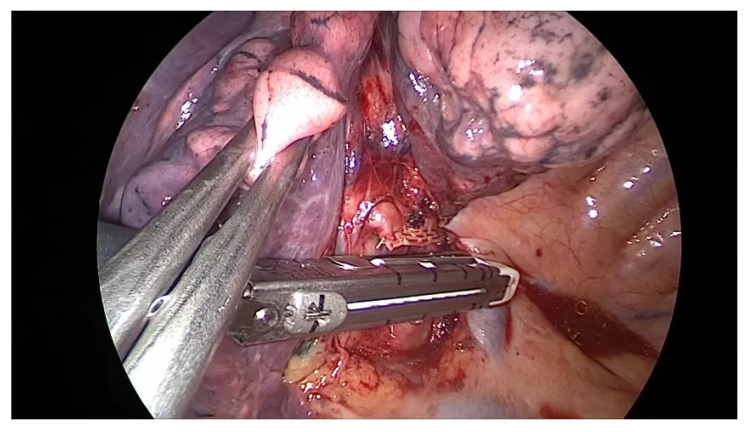
Uniportal VATS right upper lobectomy for centrolobar metastasis—stapling the right upper lobe venous drainage. VATS = Video-Assisted Thoracic Surgery.

The lobectomy might be indicated in central or multiple lesions occupying the same lobe. Pneumonectomy should be avoided because it massively impairs respiratory functions and should be performed only in highly selected patients undergoing multidisciplinary team management [1,5]. In our study, we did not perform pneumonectomy for lung metastases. Lobectomy (Figure 8) or segmentectomy was performed in 11 patients—from which in two cases wedge pulmonary resections were added, and in another two cases other anatomical structures were removed (atrial and pericardial resection in one case, and parietal pleura in another case).

Wedge lung resections were performed in 47 patients—from which in four cases an additional resection was necessary: parietal pleura (for a pleural metastasis from breast cancer), splanchnic nerve, diaphragm + pericardium and, respectively, laser pulverization of multiple millimetric lung metastases (discovered intraoperatory).

Some studies have reported better outcomes after typical resection (segmentectomy, lobectomy) [24,27]. The lower recurrence rate may be due to wider resection margins. The improved overall survival (OS) or disease-free survival (DFS) could be explained by the excision of the regional lymphatics in anatomical resection. In our study, the wedge resection patients present a lower risk of death compared with patients with lobectomy—the estimated mean survival in patients with typical (anatomical) lung resection vs. wedge (non-anatomical) was 16.5 months vs. 17.96 months; the difference presents no statistical significance. We connect this result to the smaller dimensions of metastases resected by non-anatomical resection compared to bigger metastases excised by lobectomies.

The lymph node approach in pulmonary metastasectomy is an ongoing debate in oncologic thoracic surgery [28,29,30,31,32,33,34,35,36,37,38,39,40,41,42]:

Arguments favoring lymphadenectomy in pulmonary metastasectomy are even single metastase may develop loco-regional lymph node metastasis; CT-scan may describe false-negative lymphatic metastasis (17% of cases); mediastinal lymphadenectomy presents low morbidity and mortality; can be performed minimally invasive, not only by thoracotomy; lymph node metastasis is an important negative prognosis factor and guides postoperative management. The arguments against lymphadenectomy are that it produces important adhesions which bring high difficulties and risks for ulterior surgical procedures; lymphadenectomy does not improve overall survival in patients with pulmonary metastasectomy.

Recent recommendation favor lymphadenectomy based on the “Expert Consensus Document on Pulmonary Metastasectomy” from 2019 [5]. In our study, the lymphadenectomy was performed in 46.5% of patients; lymph node metastases were found in 11.11% of cases. Lymphadenectomy was performed on a regular basis after lobectomies and occasionally after wedge resections, based on the characteristics of the lesion and the surgical team intraoperatory decision. There is no statistical difference in survival comparing the presence (12 months OS of 88%) or absence (12 months OS of 94%) of lymphadenectomy. As future “homework”, longer follow-up and a bigger number of patients would be necessary for stronger results.

The number of metastases does not construe a contraindication for pulmonary metastasectomy but a high number is associated with a poor prognosis [1,43,44]. Our results confirm these findings—the best survival was obtained after resection of 1 or 2 metastases—96% survival at 24 months; for 3 metastases excised the survival is 65% at 36 months; worst survival is for 4 metastases: 70% at 12 months and 35% at 24 months.

The median follow-up after pulmonary metastasectomy was 13 months. The median OS was not reached. The mean OS was 17 months. Estimated OS at 6 months is 96%, at 12 months is 92% and at 18 months is 87%. 

Estimated overall survival from the cancer diagnosis in our patients with pulmonary metastasectomies is 5 years median OS is of 90%, with median follow-up for cancer survival of 40 months; the median OS is 149 months. These results are encouraging considering that the patients are stage IV and more than that, at the time of diagnosis of primary cancer, 14 patients out of 58 (24.14%) were in the metastatic stage. For comparation, Chen and colab. published in 2021 a mean value of 4% of patients presenting synchronous lung metastasis at diagnostic (total 100,751 patients in 5 years), with values ranging from 0.5% (of all prostate cancers) to 13% (of all primary lung cancers) [45].

The main limit of the study is the small number of patients included, thus preventing them from drawing of definitive conclusions; another limitation of our study is the median follow-up of 13 months after surgical intervention. Still, the conclusions are valuable in guiding clinical activity and further research. Longer follow-up and prospective randomized trials are needed, with an accent on the lymph node approach in lung metastasectomy and on the comparison between surgical and non-surgical approaches to lung metastases.

## 5. Conclusions

Regarding pulmonary metastasectomy, there are practically no randomized controlled trials. Our retrospective study concludes that patients with one or two resected metastases present better survival compared to patients with more than two metastases. Patients with sarcoma metastasis present the worst prognosis and colorectal and pulmonary metastasis present the best prognosis. The non-anatomic type of lung resection (wedge pulmonary resection) may offer better survival than anatomic resections (lobectomy, segmentectomy), probably due to the smaller metastases resected. Dimensions smaller than 20.5 mm is a positive prognostic factor, emphasizing the importance of the accurate selection of patients and early referral to thoracic surgery, when pulmonary metastasectomy is indicated.

## Figures and Tables

**Table 1:** The distribution of the patients with pulmonary metastases resected regarding their underlying malignancy.

Primary Cancer	Number of Cases	Percent from Total (%)
colon	14	24.14
breast	10	17.24
lung	8	13.79
sarcomas	8	13.79
cervix	5	8.62
renal	4	6.9
endometrium	3	5.17
GIST	2	3.45
pharynx	1	1.72
pancreas	1	1.72
hepatic	1	1.72
urothelium	1	1.72
Total	58	100

**Table 2:** Type of surgical resection of the pulmonary metastases and the associated structures excised for radical surgery.

Lobe/Type of Resection	Anatomical Resections (Typical)	Associated Resections	Wedge Resection (Non-Anatomical, Atypical)	Associated Resections
Right upper lobe (RUL)	2	-	8	-
Right middle lobe (RML)	2	+2 wedge lung resections	-	-
Right lower lobe (RLL)	4	+Atrial resection, pericardiectomy, pericardioplasty	21	+Resection of diaphragm and pericardium
Left upper lobe (LUL)	2	-	18	-
				+Excision of nodule of parietal pleura
Left lower lobe (LLL)	2	+Parietal pleura	15	+Splanchnicectomy
				+Laser vaporization of millimeric lung nodules

Note: In some patients a combination of surgical procedures was necessary, resulting in a bigger number of surgical procedures compared to the number of patients.

## Data Availability

Not applicable.
